# Cultural transmission and ecological opportunity jointly shaped global patterns of reliance on agriculture

**DOI:** 10.1017/ehs.2020.55

**Published:** 2020-10-26

**Authors:** Bruno Vilela, Trevor Fristoe, Ty Tuff, Patrick H. Kavanagh, Hannah J. Haynie, Russell D. Gray, Michael C. Gavin, Carlos A. Botero

**Affiliations:** 1 Department of Biology, Washington University in Saint Louis, St Louis, MO, USA; 2 Instituto de Biologia, Universidade Federal da Bahia, Salvador, Bahia, Brazil; 3 Ecology, Department of Biology, University of Konstanz, Konstanz, Germany; 4 Department of Biology, McGill University, Quebec, Canada; 5 Department of Human Dimensions of Natural Resources, Colorado State University, Fort Collins, CO, USA; 6 Department of Linguistics, University of Colorado at Boulder, Boulder, CO, USA; 7 Department of Linguistic and Cultural Evolution, Max Planck Institute for The Science of Human History, Jena, Germany

**Keywords:** Biogeography of human agriculture, cultural evolution, comparative studies of human culture, spread of human culture

## Abstract

The evolution of agriculture improved food security and enabled significant increases in the size and complexity of human groups. Despite these positive effects, some societies never adopted these practices, became only partially reliant on them, or even reverted to foraging after temporarily adopting them. Given the critical importance of climate and biotic interactions for modern agriculture, it seems likely that ecological conditions could have played a major role in determining the degree to which different societies adopted farming. However, this seemingly simple proposition has been surprisingly difficult to prove and is currently controversial. Here, we investigate how recent agricultural practices relate both to contemporary ecological opportunities and the suitability of local environments for the first species domesticated by humans. Leveraging a globally distributed dataset on 1,291 traditional societies, we show that after accounting for the effects of cultural transmission and more current ecological opportunities, levels of reliance on farming continue to be predicted by the opportunities local ecologies provided to the first human domesticates even after centuries of cultural evolution. Based on the details of our models, we conclude that ecology probably helped shape the geography of agriculture by biasing both human movement and the human-assisted dispersal of domesticates.

## Graphical abstract

**Figure:**
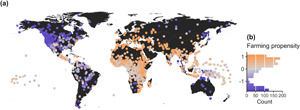

**Social Media Summary:** Ecological forces shaped the geography of agriculture by biasing human movement and the dispersal of domesticates.

## Introduction

Throughout the early and middle Holocene (12,000–4,000 BP), humans began a series of independent attempts at plant and animal domestication in up to 19 areas of Asia, Africa, Oceania and the Americas (Larson et al., 2014). These efforts eventually improved the food security of human groups, increased sedentarism and led to more reliable sources of valuable materials (P. Bellwood, 2001; P. S. Bellwood, 2005; Diamond, 2003; Pryor, 2004; Shennan, 2018; Weisdorf, 2005). Over time, the knowledge, technology and domesticated species required to support farming lifestyles spread outwards from these points of origin and within a few thousand years, human agriculture was solidly established as the predominant mode of subsistence of our species (Diamond & Bellwood, 2003).

The geographic spread of human agriculture appears to have been driven primarily by population expansion (i.e. vertical transmission; see P. Bellwood, 2001; P. Bellwood et al., 2007; Diamond & Bellwood, 2003; Shennan, 2018) and inter-group contact (i.e. horizontal transmission; see Hofmanová et al., 2016; Shennan, 2018). However, given that climate and biotic interactions play an undeniable role in current farming practices, it seems likely that early expansion processes were also shaped by ecological factors (see Diamond, 1999, 2002; King, 1993; Kirch, 2000; Larson et al., 2014; Olsson & Hibbs, 2005; Shennan, 2018). For example, the adoption of rice cultivation may have been more difficult in northern and western sections of Asia given their drier and/or colder climates. Similarly, the colonization of arid regions of modern Mexico and the Southwestern US may have led Uto-Aztecan lineages, by some accounts, to scale back their production of maize and to regain an almost complete reliance on hunting and gathering (P. Bellwood & Renfrew, 2003). Ecological factors could have also influenced the path and speed of the spread of agriculture by biasing population expansions (and therefore human-assisted dispersal of domesticates) into environments that more closely resembled the ancestral homelands of agricultural migrants (Wiens & Graham, 2005).

Despite the intuitive appeal of possible links between ecology and the spread of agriculture, it has been surprisingly difficult to demonstrate their existence in comparative analyses. Specifically, while some studies have found evidence of a potential role of ecology in the spread of agriculture (e.g. P. Bellwood et al., 2007; Bettinger et al., 2009; Gavin et al., 2018; Richerson et al., 2001; Scarre, 2005), others have not (e.g. Pryor, 2004). One possible reason for this discrepancy is that different ecological factors may have favoured the spread of different human domesticates. For example, it is likely that the spread of rice agriculture was biased towards wet environments, but that reliance on olive trees may have spread instead towards dry Mediterranean climates (Vaughan et al., 2008). It is therefore surprising that earlier inquiries into these processes considered that the adoption of such different agricultural practices could have favoured a single type of climate or ecological setting. For example, one of the first ecological hypotheses to be explicitly formulated on this topic posited that ‘poor’ (i.e., species depauperate) environments could have been more conducive to the adoption of agriculture because a scarcity of edible plants and animals was likely to favour the adoption of practices that could improve food security (Binford, 1968; Cohen, 1976). Similarly, a related prominent hypothesis posited that the adoption of agricultural practices could have instead been biased toward rich environments because these habitats offered more opportunities for new domestication and were presumably favourable to a greater number of plants and animals that had already been domesticated elsewhere (Hayden, 1995; Price et al., 1995; Sauer, 1969). Although these hypotheses probably apply to specific expansion cases and may even be generalizable to the entire world in our current context (i.e., now that knowledge of agriculture is universal and global access to suitable domesticates has improved), both of them fail to capture the effect of early ecological opportunities by implicitly assuming that all original domesticates favoured similar expansion paths. Rice and olive trees show us that this is not necessarily the case given that they exhibit strong preferences for environments with very different mean annual precipitation levels, and consequently, different biodiversity (for evidence of a positive correlation between annual precipitation and biodiversity, see Huston, 2012).

Here we address the potential shortcomings of earlier studies by estimating a proxy of early ecological opportunity that more explicitly captures the suitability of local environments for early domesticates without making unfounded generalizations about the ecological preferences of these species. Specifically, we use ecological niche models to estimate how many of the first 116 plants and animals domesticated by humans could potentially thrive in different parts of our planet and use these estimates to ask whether early ecological opportunities had any lasting effects in the global patterns of reliance on agriculture that we observe among early twentieth-century traditional societies. Because similar levels of reliance on farming could result from other processes as well, our models also account for the effects of cultural inheritance (vertical transmission), inter-group contact (horizontal transmission) and current access to plants and animals. Our analyses therefore differ from earlier studies in that we explicitly attempt to separate early vs. contemporary ecological effects, and in that we seek to explain a society's level of engagement in farming practices rather than its adoption, or lack thereof, of agriculture itself (i.e. a continuous rather than a categorical response variable). Given that humanity has greatly transformed global landscapes in the thousands of years since the origin of agriculture and that our societies have since become increasingly connected and capable of more easily introducing domesticates into non-native habitats, our null hypothesis is that traditional farming practices should reflect only the demands and ecological opportunities of their time (i.e., contemporary access to wild foods and/or the extent to which environments allow a greater variety of already domesticated plants and animals to be grown). Alternatively, given that cultural transmission and ecological constraints on the spread of early domesticates are likely to have played a major role in the adoption of agriculture, we hypothesize that these variables could also potentially exhibit significant effects up until the recent past. We acknowledge here that artificial selection has transformed the appearance and yield of many agricultural species and that this process may have even potentially altered the ecology of some of them. However, we also note that archaeological evidence indicates that many crops and animals are still generally grown in environments that resemble the ones in which they were originally domesticated and therefore assume that it is reasonable to estimate the potential geographic range of a given domesticate based on niche models that are informed by its current climatic preferences and the climate parameters of the time focus of interest (the latter to account for climate change). Overall, we confirm that current ecological opportunities as well as proxies for vertical and horizontal cultural transmission, are significant predictors of the geography of farming propensity. Additionally, we find evidence that the specific ecological requirements (and dispersal limitations) of the 116 first species domesticated by humans remarkably continued to influence the global patterns of reliance on agriculture among traditional societies up to the recent past.

## Methods

### Raw data

Data on the geographic location, language, and subsistence techniques of traditional cultural groups (*N* = 1,291 societies) were obtained from D-PLACE, www.d-place.org (Kirby et al., 2016). The dataset included here is specifically based on Murdock's (1967) compilation of ethnographic records collected between 1900 and 1950. The list of 116 species initially domesticated by humans was obtained from Larson et al. (2014). Occurrence records for each of those species were downloaded from the Global Biodiversity Information Facility (GBIF; www.gbif.org). The climate parameters associated with these localities were obtained from the EcoClimate dataset (www.ecoclimate.org; Lima-Ribeiro et al., 2015) for current (1950–1999) and historical (1900–1949) periods using the Atmosphere–Ocean General Circulation Model CCSM4, at 0.5 × 0.5° resolution.

### Integrating farming data into a continuous numerical scale

**Figure 1.**
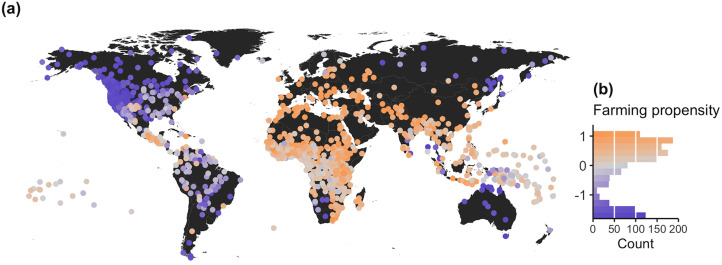
Global distribution of reliance on farming practices among traditional human societies at the onset of the 20th century. (a) Geographic location (dots on map) and mean PC1 scores (dot colour) of societies in our sample. (b) Frequency distribution of farming propensity values, ranging from heavy reliance on hunting, gathering, or fishing (blue) toward increasing dependency on agriculture and/or pastoralism (brown).

Murdock (1967) characterized the dependence of each society on hunting, gathering, fishing, agriculture and pastoralism with a categorical scale that ranged from 0 to 9: 0, 0–5%; 1, 6–15%; 2, 16–25%; 3, 26–35%; 4, 36–45%; 5, 46–55%; 6, 56–65%; 7, 66–75%; 8, 76–85%; 9, 86–100%. To account for uncertainty in the actual use of different subsistence techniques, we stochastically sampled 1,000 sets of percentage values from these categorical ranges while making sure that the total for each society summed up to 100%. We then applied a principal component analysis (PCA) for compositional data (Aitchison & Greenacre, 2002) to each of these randomly drawn sets (see Figure S1) using the R package ‘compositions’ (van den Boogaart & Tolosana-Delgado, 2008). After confirming that every PCA resulted in qualitatively similar components, we characterized each society's farming propensity as its average PC1 score across the 1,000 estimated PCAs (Figure 1). The first component in these PCAs captured 76.9 ± 0.008% of the variance in subsistence data, yielding higher scores for societies with greater reliance on agriculture and pastoralism (Figure 1b). One of the main benefits of characterizing farming propensities on a continuous scale is that it prevents loss of information in cases of partial implementation or combined practices, which were common in the Americas, across sub-Saharan Africa and in the Pacific Islands (Figure 1a).

### Ecological niche models

Geographic bias in the propensity to report species sightings could potentially affect the results of ecological niche modelling. To minimize such biases, we overlaid all occurrence records from GBIF on a 0.5 × 0.5° global grid and kept only one randomly selected record per cell. All species with fewer than 15 records were subsequently removed from the analysis (*n* = 9). From the 19 bioclimatic variables available in EcoClimate, we selected a set that captured a meaningful range of factors that could potentially influence species distributions while minimizing collinearity: annual mean temperature, temperature annual range, precipitation of wettest month, precipitation of driest month, and precipitation of warmest quarter. We used the maximum entropy (Maxent) approach to model ecological niches (Phillips et al., 2006) as implemented in the R package ‘dismo’ (Hijmans et al., 2017). Models were estimated allowing all Maxent features (Linear, Quadratic, Product, Threshold and Hinge) in order to capture any potentially complex relationship between species occurrences and climate. Additionally, the regularization multiplier in ‘dismo’ was set to 1 to avoid model overfitting. Every niche model was estimated using a training set of 70% of occurrence records for a given focal species and was subsequently cross-validated with the remaining testing set. Cross-validation involved calculating the area under the curve (AUC) of the plot of correct vs. falsely predicted occurrences for the 30% of data that had not been used to inform the model (i.e. the Receiver Operating Characteristic curve). Values of AUC range from 0 to 1, and values above 0.7 are considered to indicate significantly better performance than chance. All ecological niche models generated here yielded AUC values above 0.9.

Because occurrence data for domesticated species reflect current distributions, we generated and cross-validated every niche model with current climate values (1950–1999). The typical output of an ecological niche model is an equation that allows one to calculate the percentage suitability of any given site for a species of interest. To generate predicted distributional ranges for early domesticates that matched the same time period in which cultural data were collected (Kirby et al., 2016; Murdock, 1967), we therefore generated these predictions by computing suitability using local climate data from 1900 to 1949. A given map cell was included in the expected distribution of a domesticate for that time period if its suitability value equalled or exceeded the minimum suitability value observed among known localities of record (i.e., we used a non-omission threshold). Presence–absence maps for the 105 modelled species that met our threshold of occurrence records were subsequently combined to produce a global map of the number of early domesticates that local environments could support at a 0.5 × 0.5° resolution.

It is also possible that dispersal constraints limited early access to domesticated species (Barve et al., 2011; Zeder et al., 2006). For example, perfectly suitable regions in continents other than the continent of origin may not have come into contact with a given domesticate until relatively recently. To investigate these limits on early ecological opportunity, we restricted the range of potential availability for each early domesticate to only suitable map cells within a given distance from their corresponding points of origin (see Larson et al., 2014). Because the actual magnitude of dispersal constraints is currently unknown, we took a data-driven approach to set this parameter for downstream analyses. Specifically, we generated a series of richness maps with different dispersal constraints and chose the value that maximized the Pearson's correlation coefficient between the farming propensities derived from PCA and the number of predicted early domesticates available for each society (see ‘Statistical analysis’ and Figure S2). The range of dispersal constraints we explored went from 10 to 40,000 km (i.e., the circumference of the entire world as measured at the equator), with 10 km increments. The dispersal constraint that best predicted farming propensities was 8,000 km, which roughly translates to dispersal processes that are limited to a continental scale.

### Cultural modes of transmission

We estimated the potential for vertical and horizontal transmission of agriculture based on geographic proximity, language and cultural similarity. Given the current absence of a widely accepted global phylogeny of languages, we used instead the language family groupings from Glottolog (Hammarström et al., 2016) to account for non-independence issues directly related to cultural phylogenetic relationships, i.e., vertical transmission (see Botero et al., 2014). The potential for horizontal transmission of farming was estimated as the average farming propensity value of the *k* closest neighbours of each society. To ensure that our metric of horizontal transmission best captured the spatial structure of the dataset, we chose a *k* value that maximized our ability to account for the spatial autocorrelation in the residuals of the correlation between horizontal transmission estimates and farming propensities (i.e., *k* = 7 neighbours).

### Statistical analysis

All of the analyses reported here were performed in R version 3.3.2 (R Core Team, 2016) and are based on the subset of 1,118 societies for which data are available for all covariates in our model (code available at https://github.com/BrunoVilela/Farm_Ecology). Estimates of contemporary ecological opportunities were derived from global maps of vascular plant richness (Kreft & Jetz, 2007) and mammal richness (compiled from shapefiles provided by the 2010 IUCN Red List of Threatened Species v. 2010.4; available at: www.iucnredlist.org; downloaded 17 April 2012). Although these richness values are loosely correlated with the potential availability of early domesticates (Pearson's product–moment correlation coefficient: with mammal diversity *r* = 0.51; with plant diversity *r* = 0.22), they are included here to capture local access to wild sources of food and/or local opportunities to grow or domesticate a greater variety of plants and animals. We began by estimating independently the effects of all possible combinations of potential for horizontal transmission, potential for vertical transmission, current ecological opportunity (i.e., local vascular plant richness and mammal richness) and the ecological opportunity for early expansion processes (i.e., environmental suitability for the set of 116 first human domesticates) on farming propensity. Subsequently, we used this set of models to partition the proportion of variance explained by individual predictors and combinations of predictors (Peres-Neto et al., 2006). We note that, although Language family was treated as a random effect in models reported in Table 1, it was defined as a fixed effect in the variance partition analysis in order to make the *R*^2^ values of models with and without this term comparable. To evaluate whether the fully parameterized model had successfully accounted for spatial autocorrelation in our data, we generated a Moran's I spatial autocorrelogram on the residuals using the R package ‘letsR’ (Vilela & Villalobos, 2015) and 12 distance classes with equal sampling levels. The spatial autocorrelogram of our model residuals shows that, although farming propensities are highly spatially structured, we have successfully accounted for spatial autocorrelation (i.e., Moran's *I* values are close to zero in all distance classes, see Figure S3).

**Table 1.** Linear mixed models of farming propensity in early twentieth century traditional societies as predicted by neighbourhood effects (i.e. horizontal transmission), the number of early domesticates capable of thriving under local climatic conditions (i.e. historical ecological opportunity) and the current mammal and vascular plant diversity (i.e. current ecological opportunity). Phylogenetic non-independence is accounted for by including language family as a random effect. The upper and lower halves of the table respectively summarize our findings based on a model with and without dispersal constraints (i.e., 8,000 km radii from corresponding centres of origin)

Model	Parameter	Standardized coefficient	d.f.	*t*	*p*
With dispersal constraints	Intercept	−0.148 ± 0.039	84.125	−3.754	<0.001
	Potential number of early domesticates	0.073 ± 0.029	1097.288	2.504	0.012
	Mammal diversity	−0.043 ± 0.026	1064.815	−1.658	0.098
	Vascular plants diversity	0.051 ± 0.024	774.493	2.155	0.031
	Neighbourhood effect	0.655 ± 0.033	531.076	19.838	<0.001
	*Estimated variance ± SD for language family (random effect): 0.120 ± 0.346*				
Without dispersal constraints	Intercept	−0.151 ± 0.040	84.000	−3.740	<0.001
	Potential number of early domesticates	0.025 ± 0.022	1022.235	1.105	0.269
	Mammal diversity	−0.023 ± 0.024	986.055	−0.949	0.343
	Vascular plants diversity	0.043 ± 0.025	800.415	1.728	0.084
	Neighbourhood effect	0.689 ± 0.028	317.561	24.200	<0.001
	*Estimated variance ± SD for language family (random effect): 0.129 ± 0.360*				

## Results

**Figure 2.**
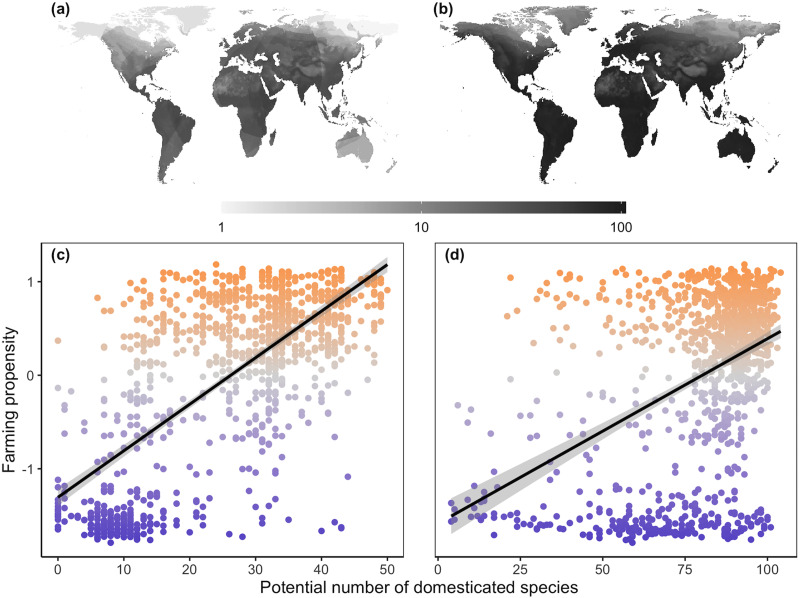
Alternative algorithms for estimating global variation in the suitability of local climates for the first 105 species domesticated by humans (a and b) and their effect on farming propensity (c and d). (a) Number of domesticated species expected to be available at each site given ecological conditions and a dispersal limit from corresponding centres of origin of 8,000 km. (b) Predicted number of early domesticates without any dispersal constraints. (c) Effect of (a) on the farming propensity of early 20th century traditional societies. (d) Effect of (b) on the farming propensity of early 20th century traditional societies.

Figure 2a depicts the predicted availability of early agricultural domesticates at the beginning of the twentieth century as generated through ecological niche modelling and a dispersal range cut-off of 8,000 km. As expected, ecological suitability for the initial set of human domesticates is generally highest in tropical and subtropical environments. It is noteworthy that when dispersal constraints are considered (compare Figure 2 with Figure 2b), our niche models predict relatively low access to early domesticates in currently highly productive agricultural regions that nevertheless tend to harbor only hunter–gathering traditional societies (i.e., east and west Australia and the Central Valley of California, USA).

**Figure 3.**
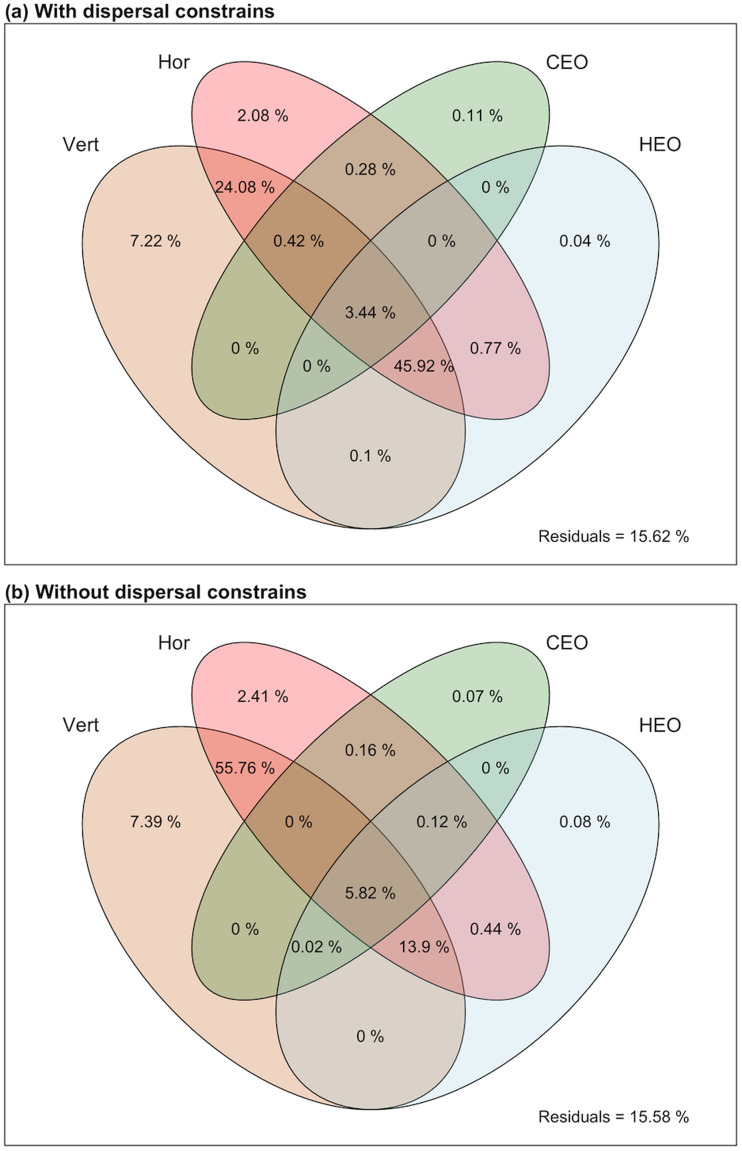
Variance components in our models of farming propensity among traditional societies in the early 20th century. Model variants that either considered (a) or not (b) dispersallimitations are depicted separately. Hor = horizontal cultural transmission; Ver = vertical cultural transmission; CEO = current ecologicalopportunity; HEO = historical ecological opportunity.

Alternative versions of our model of farming propensity with and without dispersal constraints are summarized in Table 1. In general, our analyses indicate that early twentieth-century farming practices were shaped by recent ecological opportunity (i.e., local richness of plants and animals) and by both the vertical and horizontal transmission of culture (as estimated from language family and neighbourhood-level reliance on farming). Additionally, we find that even after accounting for these effects, twentieth-century reliance on farming continues to be significantly predicted by how suitable local environments were for the specific set of first plants and animals domesticated by humans. Two aspects of this finding deserve special attention. First, most of the variance explained by this index of early ecological opportunity is jointly explained with cultural transmission (Figure 3), and second, the effects of early ecological opportunities are stronger when imposing continental-level dispersal limits on the early spread of domesticates (as seen in the 2.6-fold reduction in *R*^2^ between Figures 3a and 3b).

## Discussion

Our analyses partially reconcile different mechanistic views on the spread of agriculture that have been largely presented as antithetical. Specifically, we find that of our proxies for both the vertical and horizontal transmission of culture predict geographic patterns of farming propensity among traditional societies in the early twentieth century (Table 1 and Figure 3). Much debate still exists regarding the relative roles of vertical and horizontal transmission in the early spread of agricultural subsistence (Shennan, 2018). Some researchers have suggested that vertical transmission may have dominated this process, arguing that agriculture spread primarily through the movement of agriculturalists into new territories (e.g. P. Bellwood, 2001; P. Bellwood et al., 2007; Diamond, 2003). Other researchers have instead posited that cultural diffusion may have been the stronger mechanism, facilitating the horizontal spread of agricultural knowledge and domesticates between cultural groups (Edmonson, 1961; Hofmanová et al., 2016). The extensive overlap in variance explained by vertical and horizontal transmission in our model (84.37%) suggests that it is risky, at this point at least, to attribute greater importance to either one of these processes. However, given that our proxies for both of these processes explain unique, even if small, aspects of the total variability, we can at least conclude that both are likely to have shaped the spread, adoption and continued evolution of agricultural practices.

Our findings also demonstrate that the recent geography of agriculture was shaped not only by contemporary ecological factors but also by ecological biases in early expansion processes. As expected from the fact that most of the world had some level of exposure to agriculture by the early 1900s, our proxies for contemporary ecological opportunities (i.e., local richness of wild plants and animals) are correlated with farming propensity. Specifically, we find a stronger reliance on farming in richer environments, suggesting that the adoption of farming is more strongly favoured by opportunity (i.e., environments that can potentially sustain a greater diversity of domesticated plants and animals) than food insecurity, a pattern that has also been shown to be the case for the timing and locations of agricultural origins (Kavanagh et al., 2018).

Additionally, we find that, even after accounting for the effects of cultural transmission and contemporary ecological opportunity, there is still a significant effect of the degree to which local environments were specifically suitable to the set of 116 first plant and animal domesticates. We interpret this finding as an indication that ecology may have influenced the geography of agriculture by biasing the spread of domesticates because our models are more informative when dispersal constraints are considered (Table 1) and because a majority of the variance explained by our proxy of early ecological suitability is jointly explained with vertical transmission (Figure 3). Given that humans are the primary vectors of dispersal for most domesticated species, an ecological bias in human-assisted dispersal is further supported by earlier findings that cultural migrants exhibit a preference for settling in familiar environments (Wiens & Graham, 2005).

Early adoption of certain crops and animals could have also subsequently biased a society's reliance on agriculture by facilitating or hindering the adoption of other domesticates. For example, it is possible that the adoption of new domesticates was biased toward species that do not require major changes to a society's agricultural infrastructure and practices. Alternatively, it is also possible that the identity of domesticates is not the driving force for this effect. Specifically, ecological similarity could instead have facilitated the transfer of knowledge (as opposed to the movement of people and domesticates) and in doing so, it may have enabled the spread of agriculture by helping initiate new local processes of domestication (see large overlap in the variance explained by horizontal transmission and early ecological suitability in Figure 3). It should be noted though, that even this latter possibility is constrained by human movement because prehistoric transfers of knowledge between groups would presumably require some level of person-to-person contact.

The complex interaction between human action and ecology suggested by our findings may also provide a viable explanation for why perfectly suitable habitats remained largely devoid of agriculture until relatively late in history. For example, traditional societies in the coastal regions of Australia and the Central Valley of California exhibit low reliance on agriculture (Figure 1) even though these environments are among the most productive agricultural areas of the present time (e.g. California currently produces 8% of the entire agricultural output of the US even though it contains less than 1% of its total farmland: California Department of Food and Agriculture, 2015). The predictions from our niche models indicate that even in the absence of recent improvements in irrigation and soil quality, the local climates of these regions would have easily accommodated the growth and well-being of many of the first species domesticated by humans in other parts of the world (Figure 2b). However, when dispersal constraints are taken into account (Figure 2a), we see instead that a much lower number of early domesticates were likely to be available in these regions, indicating that local climates were considerable less suitable for species that happened to be domesticated nearby than for those that were domesticated 8,000+ km away. It is therefore possible that traditional Californian and Australian societies did not develop a strong reliance on agriculture simply because the geographic reach of their network of contacts with outside societies was only large enough to grant them access to early domesticates that could not thrive in their own land. For example, the main crops developed near California (i.e., maize, bean and squash) are heavily reliant on summer rainfall, which makes them unsuitable for the dry summers of the Central Valley (captured by differences in the mean precipitation of the lowest quarter in our dataset). Australia's adoption of agriculture may have been similarly thwarted by a mismatch between its climate and the domesticates that were developed nearby in wetter habitats (e.g., yams, taro or bananas), and could have also been further prevented by the infertility of its heavily weathered soils (Grundy et al., 2015), an important ecological factor not considered in our study.

In conclusion, our findings indicate that geographic variation in reliance on agriculture among traditional societies in the early twentieth century can be consistently predicted by language family, the agricultural practices of near neighbours, current access to wild plants and animals, and the degree to which local environments specifically favoured the initial set of human domesticates that were developed less than 8,000 km away. Given the large fraction of variance that is jointly explained by different combinations of these variables, we strongly caution against simplisticaly interpreting the size of individual variance components in our models as possible indicators of their relative importance. Additionally, we highlight that, in aggregate, our findings remind us that no matter how technologically advanced our species has become, our subsistence is still solidly anchored in both history and its ecological context.

## Supplementary material

For supplementary material accompanying this paper visit http://dx.doi.org/10.1017/ehs.2020.55. click here to view supplementary material

## Research transparency and reproducibility

The data that support the findings of this study are openly available in D-PLACE at www.d-place.org, the Global Biodiversity Information Facility GBIF at www.gbif.org, EcoClimate at www.ecoclimate.org, and the IUCN Red List of Threatened Species v. 2010.4 at: www.iucnredlist.org.
