# Supplementary figures and images for: Cultural transmission and ecological opportunity jointly shaped global patterns of reliance on agriculture
Source: Evol Hum Sci. 2020 Oct 26;2:e53. doi: 10.1017/ehs.2020.55 (PMC10427461; doi:10.1017/ehs.2020.55)

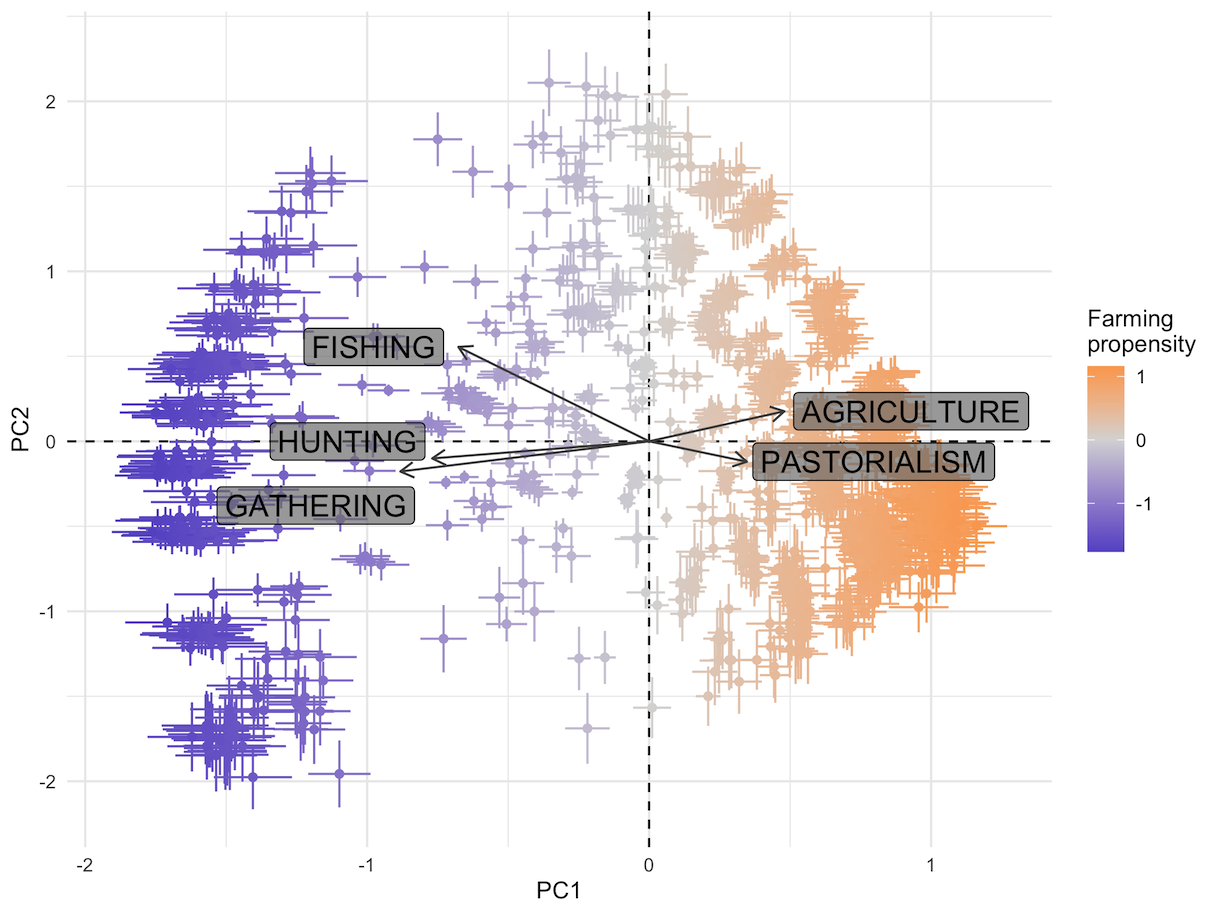

Supplement: Supplementary file 1 [file ehssup.zip › S2513843X20000559sup001.tiff]

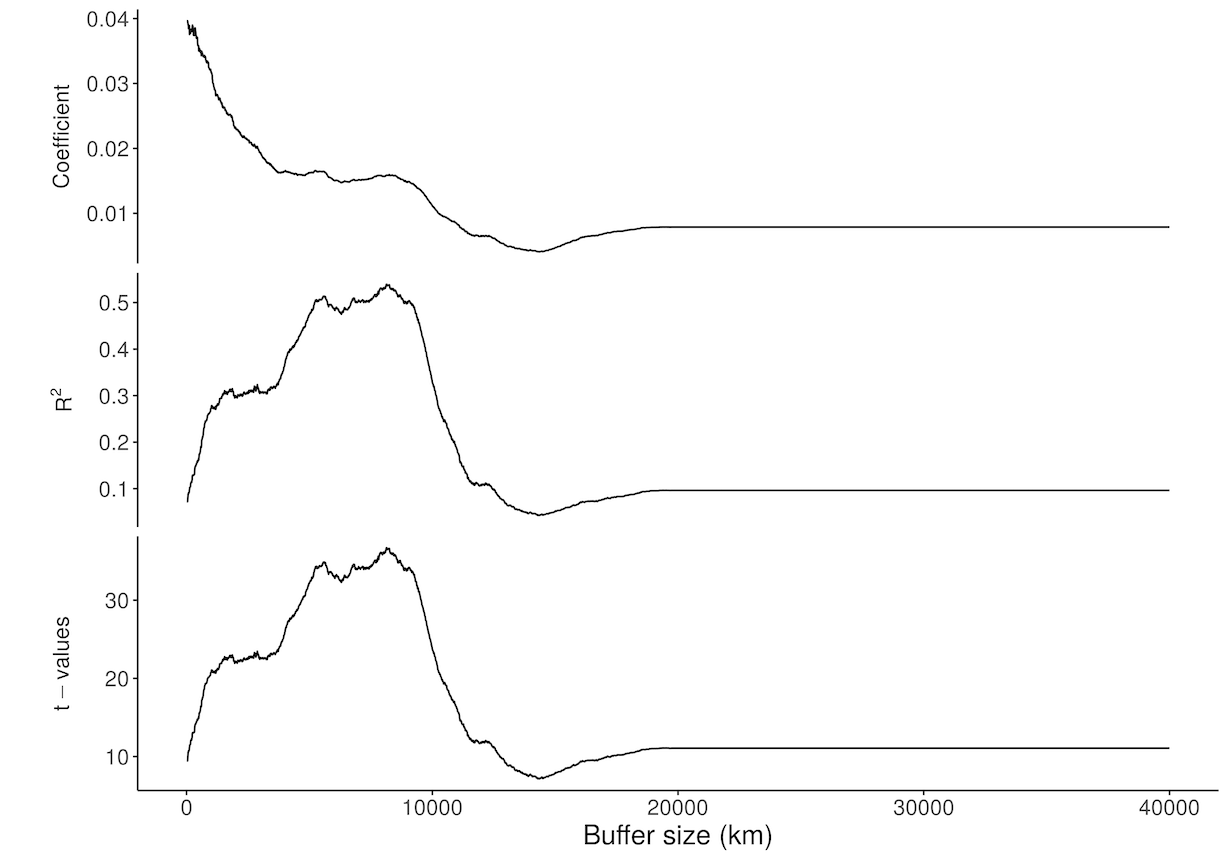

Supplement: Supplementary file 1 [file ehssup.zip › S2513843X20000559sup002.tiff]

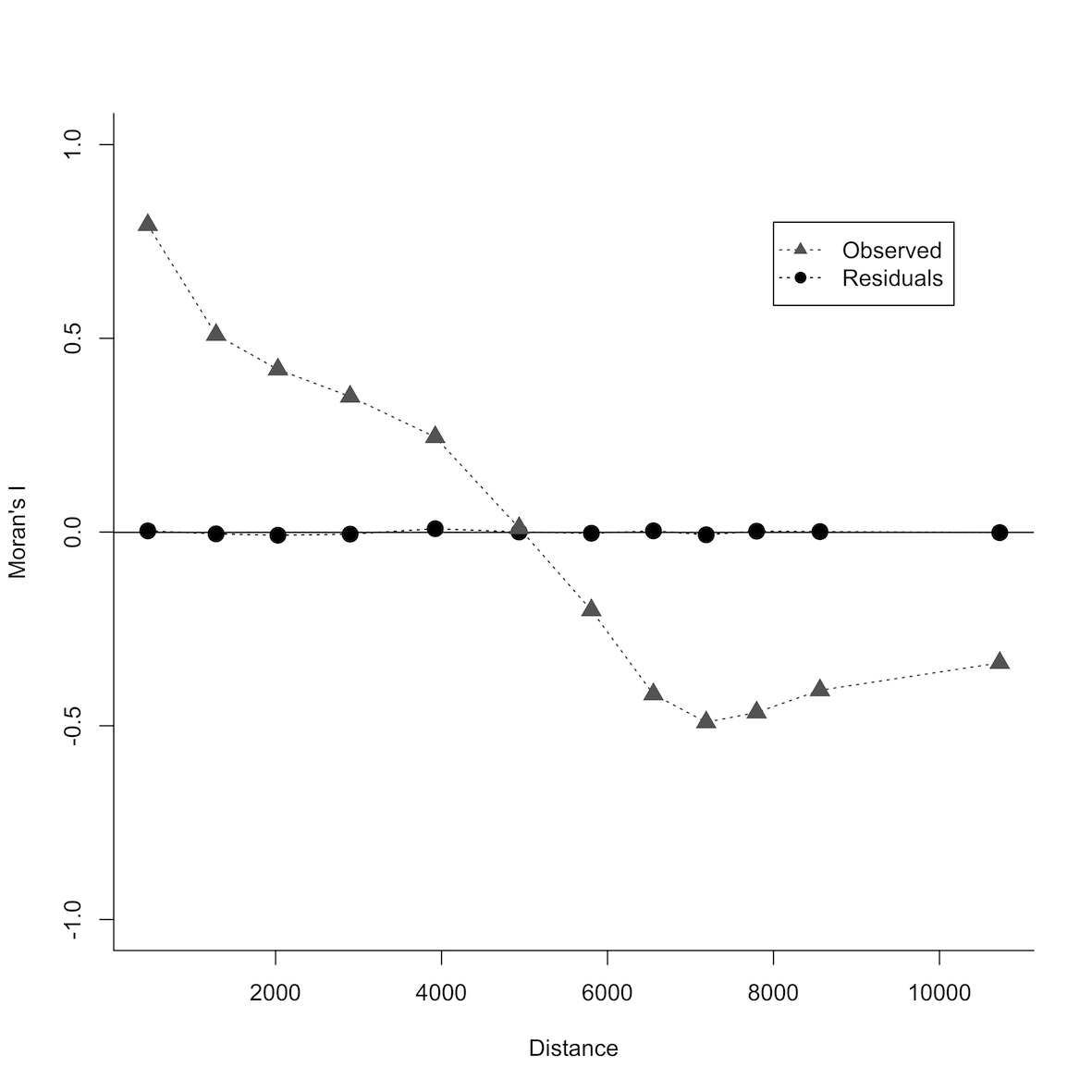

Supplement: Supplementary file 1 [file ehssup.zip › S2513843X20000559sup003.tiff]
